# The impact of a school backpack's weight, which is carried on the back of a 7-year-old students of both sexes, on the features of body posture in the frontal plane

**DOI:** 10.1186/s13102-022-00448-8

**Published:** 2022-04-02

**Authors:** Mirosław Mrozkowiak, Marta Stępień-Słodkowska

**Affiliations:** 1 Physiotherapy Practice AKTON, Warsaw, Poland; 2 Faculty of Physical Culture and Health, Institute of Physical Culture Sciences, University of Szczecin, Al. Piastów 40B blok 6, 71-065 Szczecin, Poland

**Keywords:** Children’s health, Moire topography, Physical fitness, Postural asymmetry factor

## Abstract

**Background:**

The lifestyle of children has a significant impact on the future health of the whole society. Therefore, health education, prevention and monitoring of health determinants is important at every stage of ontogenesis. This requires a thorough knowledge of the schoolchild's environment, perceived as a wide set of stressors, including not only genetic but also epigenetic factors. One of them is the issue of the correct and abnormal body posture at school and on the way there. The goal of the study was to show the influence of the weight of the back carried container with school supplies on body posture.

**Method:**

The research was carried out as part of a project that examined the impact of carrying weights, which are school supplies, by children on their posture. The research material consisted of data obtained from a group of 65 students (35 girls, 30 boys) aged 7 years. Body posture tests were carried out, using the projection moiré method in 4 positions: 1-habitual posture, 2-posture after 10-min of asymmetric axial load, 3-a posture after 1 min of the load removal, 4-a posture after two minutes of the load removal. Physical fitness was measured with the Sekita test. The obtained data were statistically analyzed.

**Results:**

The significance of differences between the 1st and 2nd measurements was analyzed to determine the impact of the backpack load and the correlation with physical fitness, and to study its influence on the value of the differences in posture features. Considering the differences in the volume of posture features among boys between the 1st and 2nd measurement, the Wilcoxon’s rank test showed a statistically significant difference in the range of all analyzed variables, except for the torso bend angle to the right (KNT+), where no statistically significant change was noted. A statistically significant difference in the volume of all analyzed variables was observed among the girls.

**Conclusions:**

Carrying school supplies on the back induces significant changes in the value of the features describing the body posture in the frontal plane. The greater weight of the container and carrying time, and intensity of physical effort is the greater the changes will be. Physical fitness has a various and sex-dependent influence on the value of changes in body posture features because of carrying school supplies. Among boys it significantly affects the asymmetry of the torso bend, shoulder height, the waist triangles height and width, whereas among girls it affects the asymmetry of the shoulders and the distance of the angles of the lower shoulder blades from the line of the spinous processes of the spine. Among boys the changes in the value of posture features are mostly influenced by endurance and speed, but strength, power and agility are of lower influence, whereas among girls only agility matters.

## Background

The student's environment plays a significant role in the development of biomechanical disorders of body posture. Both school and home should support the student in their pursuit of cultivating a healthy lifestyle according to Cendrowski’s set of ten principles [1]. One of the strategic goals of the project of the National Health Program in Poland in 2007–2015 was to "reduce premature morbidity and reduce the negative effects of chronic diseases of the articular system". In order to achieve this goal, the work, study and leisure environments were modified to promote health. The determinants of health are well described by the French concept from 1991, which divides them into six groups: natural (geographic) environment, demographic, socio-economic, psycho-cultural, political and administrative, related to the organization and functioning of the health care system. In the case of a student, these will be: a sedentary lifestyle, inadequate position during study and rest, improperly carried and too heavy school supplies. There is a great need for movement, defined as the first peak of motor skills in children aged 7–9 years [2]. Movement during this time is the greatest physiological need of the developing organism [3]. Inadequate activity may increase the risk of overweight and obesity. It also causes abnormalities in the development of muscle and bone tissue, which in turn causes posture defects. Other factors influencing the development of posture defects are: adopting incorrect postures during rest, unsuitable places for studying at school and at home, and wearing overloaded school bags. The lifestyle is dominated by activities that do not require effort [4, 5]. Hong et al., after measurements carried out in a group of 410 boys aged 10 years, looked for relationships between the quality of body posture and the weight of the backpack carried without a load with 10%, 15%, 20% of body weight. There were no significant dysfunctions in body posture and gait when the weight of the backpack was 10% of the body weight. However, when the weight was 15% and 20% of the body weight, there was a statistically significant increase in the torso bend angle in the sagittal plane [6]. According to Polish Chief Sanitary Inspectorate’s recommendations regarding the proper choice and manner of carrying school items, a schoolbag cannot weigh more than 10–15% of the body weight and should be carried on both shoulders. There should be a stiffened support touching the back and with equal wide straps, and the heavier items should be placed on the bottom of the backpack whereas the lighter ones higher [7]. The author's interest is due to the significant problem of postural defects in children. Although the pre-school period is characterized by high inter-individual variation, prophylaxis in shaping the correct body posture is very important already in this period [8]. This is due to the different degree of development of somatic features, varied body structure and posture, as well as changes in the musculoskeletal system. Early diagnosis and correction of posture defects allows to eliminate defects that may affect the quality of life in adulthood [9]. It is noted in the literature that the later period of the child's development (grades 1–3) is the period in which the most postural defects arise. The research showed that only 20% of the respondents did not have any postural defects [10]. The goal of the implemented program was an attempt to determine the impact of the loading weight of carried school supplies in the following way: on the left or right shoulder with the left or right hand, on the chest, on the back and chest, obliquely on the left shoulder and at the right hip, and obliquely on the right shoulder and left hip. The goal of the study was to show the impact of a school backpack's weight, which is carried on the back of a students, on the features of body posture.

Taking into account the previous considerations, the following research questions were asked:

1. Does the accepted way of carrying the school items significantly affect the value of the body posture features in the frontal plane and do these disorders depend on gender?
2. Does physical fitness show a significant relationship with the value of posture disorders and is this relationship dependent on gender?
3. Can the way of carrying school supplies be recommended to 7-year-old children?

Our own research results and the analysis of the available literature suggest that:

1. There are significant differences between the values of the features of habitual body posture and posture influenced by asymmetric load. The differences will be greater among girls than among boys.
2. In the adopted way of carrying the school supplies, deficiencies in body posture are mostly influenced by general fitness. The differences will be less visible among children with greater physical fitness, regardless of gender.
3. The adopted way of carrying school supplies weighing 4 kg is not recommended for 7-year-old children because of significant disorders in body posture features.

## Methods

In total, 65 students participated in the research, of whom 53.84% (35 people) were girls and 46.15% (30 people) were boys. Children who participated in the study were from randomly selected kindergartens of the West Pomeranian and Greater Poland voivodships. Bad body postures and disorders were not a criterion excluding the participation in the research. The research was qualified according to the scheme: if the subject was 6 years, 6 months and 1 day old and was under 7 years, he was included in the 7-year-old age group. This allowed to use the previously developed normative scopes appropriate for this age and sex category, diagnosing the quality of body posture found at the day of the examination [11].

The research was carried out in accordance with the principles of the Declaration of Helsinki, and the consent for their implementation was obtained from the student and his legal guardian, the tutor and the kindergarten management and the bioethical committee (KEBN 2/2018, UKW Bydgoszcz). The research started on the 27th of May 2019, and always were conducted from 9.00 a.m. to 2.00 p.m. in the properly prepared same room. On the first day, all children were introduced with the purpose and course of the research. The children were also encouraged to keep the anthropometric points marked with a marker pen on the skin. A preschool teacher’s assistant of the study group was always present during the measurements, to ensure the children’s emotional stability. During the research, the adopted rules of the research procedure were followed. The research was conducted by the same author. The author is a physiotherapist with many years of professional experience. He has been conducting research in this field for many years [11–14].

The Wroclaw Physical Fitness Test for 3–7-year-old children was used to diagnose the children’s physical fitness [15]. According to the author, the test has a high degree of reliability and is adequate in terms of discriminatory strength and difficulty level [16]. The proposed test consists of four trials carried out as part of the Sports Day, which significantly increased the motivation to exercise in the presence of parents: agility (pendulous run with carrying blocks at 4 × 5 m distance), strength (long jump), speed (running at 25 m distance), force (both hands overhead throw with a 1 kg ball). The author modified the test by adding a fifth attempt—endurance. Starting position—high starting stance. Movement—run at 300 m. The running race time from start to finish line was converted into points depending on the gained score and gender. If a child did not finish the race, the score was nil. The run took place on a fitness trail with a hardened surface in compliance with all safety standards [17].

One of the most objective methods for diagnosing body posture is the projection moiré method used in the research. Much research on postural defects has been conducted with the use of projection moiré method [14, 18–22]. The projection moiré method consists in using the bending of a light beam between a screen with a net and its shadow which is projected onto the tested person standing behind the screen. It is a non-invasive method with a short measurement time. It can be repeated many times without exposing the patient. The method works well in a situation where a large group of respondents is measured and the available measurement time for each person is limited. The level of accuracy and repeatability of measurement results largely depends on the accuracy of performing all elements of the measurement procedure.

**Fig. 1:**
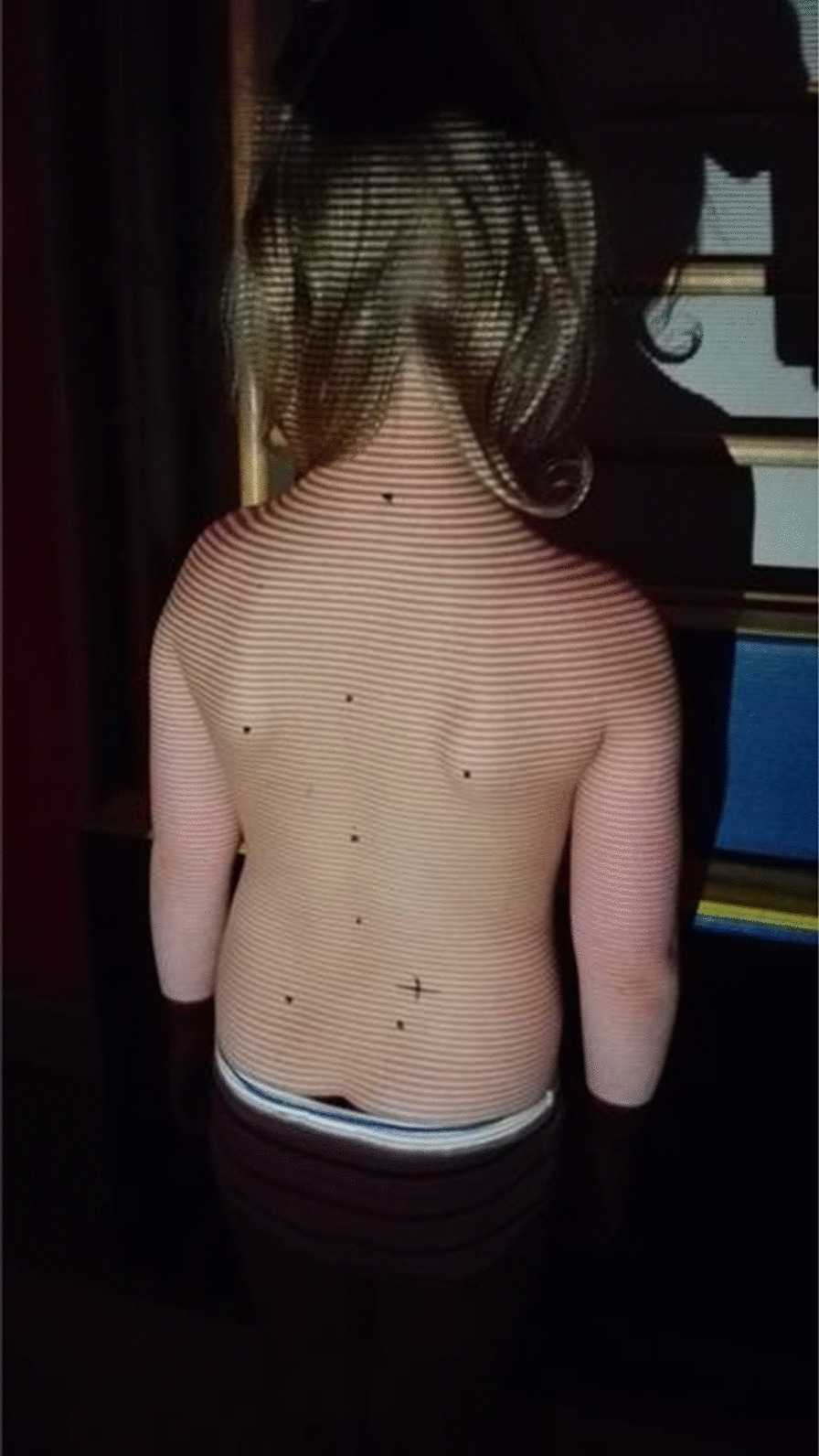
Position 1: habitual posture

**Fig. 2:**
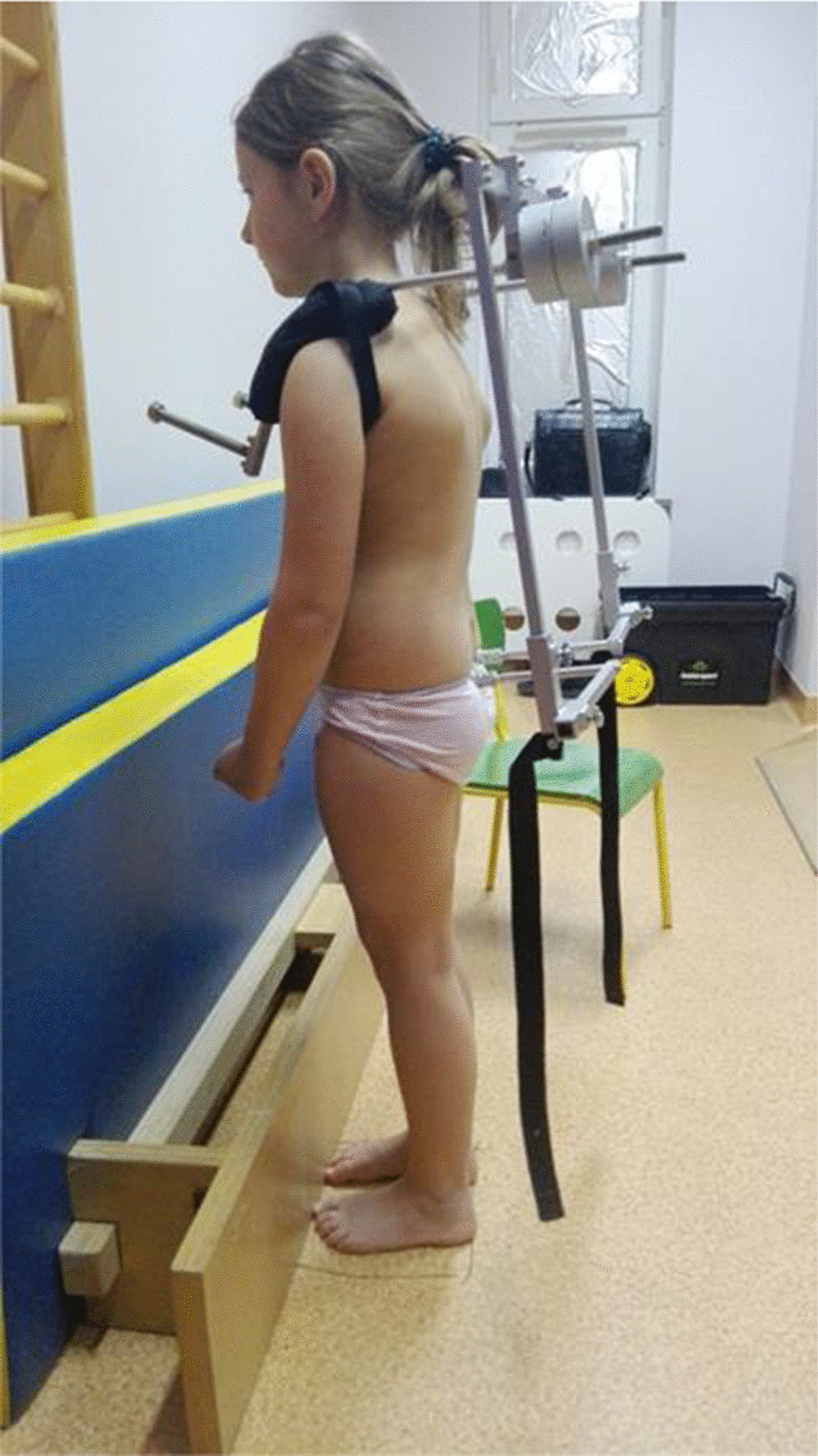
Axially asymmetric load method

Any loading of body posture was provided by the constructed diagnostic frame (utility design protection right no. W.125734). The presence of the assistant during the examination was dictated by the need to minimize the time from the load removal to second registration of the values of the posture features. Every effort has been made to ensure that the weighted frame is individually adapted to the type of a child's build. The adopted 10-min load time was the average time to go from the place of living given in the questionnaire completed by the parents [23]. On the other hand, the load mass was determined by averaging the weight of school items carried by 1st grade children from a randomly selected primary school with the burden of 4 kg. Selected features of body posture were measured in 8 positions, 4 for each way of carrying. The first position—habitual position, Fig. 1. Second position—posture after 10 min of asymmetric oblique loading (in the last 5 s). Third position—posture one minute after the load removal. Fourth position—posture two minutes after the load removal. The load was supposed to imitate the way of carrying school supplies, Fig. 2. The subject could move freely. Thereby, there were attempts made to exclude the overlapping of postural muscle fatigue from one position to another during the examination. This is in line with the results of Mrozkowiak's research, which shows that after this time the features can take the initial values [12]. The children’s height and weight as well as the weight of the carried school supplies were measured with a medical balance before the first day of the tests.

Due to the methodology of the research, the examination of a child standing on a strain gauge mat was abandoned [14].

To minimize the risk of making mistakes in the measurements of selected posture features, the test procedure described by Mrozkowiak was used [11, 12, 14].

The Wrocław fitness test made it possible to measure the strength, power, speed and agility of preschool children. The author modified Sekita's test with a test of endurance. Definitions of the examined physical and complex motor skills are generally available in the literature on the subject.

The measuring device used in the test determines several dozen features describing the body posture. Sixteen angular and linear features of the spine were selected altogether with pelvis and torso in the frontal plane (KNT, KNT− KLB, KLB− UL, UL−, OL, OL−, TT, TT−, TS, TS−, KNM, KNM−, UK, UK−) [18], as well as the body weight (Mc) and height (Wc) for statistical analysis.

The analysis of the research results was performed using the IBM SPSS Statistics 26 program.

## Results

In total, the research carried out in a group of 65 people of both sexes allowed for the registration of 5005 values of features describing body posture in a habitual posture and dynamic positions, body weight and height, and physical fitness, Table 1.

**Table 1:** Basic characteristics of the studied group

	Girls	Boys
Average body weight	24.46 kg	24.56 kg
Average body height	123.87 cm	123.00 cm
Amount	35	30

*Source*: Own research

Considering the differences in the volume of posture features among boys between the 1st and 2nd measurement, the Wilcoxon’s rank test showed a statistically significant difference in the range of all analyzed variables, except for the torso bend angle to the right (KNT+). Where no statistically significant change was noted, Table 2. A statistically significant difference in the volume of all analyzed variables was observed among the girls, Table 3. The following symbols are used in the tables: M—arithmetic mean, Me—median, SD—standard deviation, Z—Wilcoxon’s test statistic, "*p*"—Wilcoxon’s test significance. The level of significance was set at *p* < 0.05 marked as *, and additionally the significance level *p* < 0.01 marked as **.

**Table 2:** Significance of differences in the value of posture features in the frontal plane between 1st and 2nd measurement with the back loading among boys

No	Variables	Measurement 1			Measurement 2			Wilcoxon’s test	
		M	Me	SD	M	Me	SD	Z	*p*
1	DCK	308.98	314.05	22.87	290.72	292.65	21.15	− 4.782	< 0.001**
2	Alfa	8.28	8.45	1.52	4.82	4.85	2.10	− 4.783	< 0.001**
3	Beta	9.90	9.75	1.13	21.76	21.70	1.17	− 4.785	< 0.001**
4	Gamma	11.10	11.20	1.19	9.10	9.00	1.16	− 4.788	< 0.001**
5	Delta	29.28	29.65	2.45	35.69	35.40	2.70	− 4.785	< 0.001**
6	KPT−	3.74	4.15	1.34	5.72	6.40	1.59	− 3.929	< 0.001**
7	KPT+	4.40	4.75	0.69	15.43	16.50	2.32	− 2.807	0.005**
8	DKP	278.15	279.00	8.96	269.48	273.10	20.01	− 4.785	< 0.001**
9	KKP	159.04	159.00	1.55	149.14	148.90	1.63	− 4.785	< 0.001**
10	RKP	185.63	185.30	13.49	181.28	180.50	13.68	− 4.389	< 0.001**
11	GKP	20.26	19.95	1.40	15.43	15.35	1.69	− 4.786	< 0.001**
12	DLL	246.61	247.00	11.98	242.15	242.90	12.20	− 4.785	< 0.001**
13	KLL	161.82	161.95	2.22	153.42	153.35	2.52	− 4.784	< 0.001**
14	RLL	134.86	135.60	11.07	130.68	131.55	10.78	− 4.785	< 0.001**
15	GLL	23.44	24.45	3.19	32.84	32.65	3.36	− 4.788	< 0.001**
16	KNT−	1.56	1.40	1.04	2.61	2.25	1.03	− 4.116	< 0.001**
17	KNT+	2.04	2.35	1.50	3.83	4.10	1.88	− 2.524	0.012**
18	KLB−	2.60	1.90	1.64	4.31	4.00	1.31	− 2.527	0.012**
19	KLB+	1.60	1.05	1.39	3.02	2.70	1.59	− 4.112	< 0.001**
20	UL−	3.01	4.15	2.30	4.35	5.30	2.29	− 2.527	0.012**
21	UL+	2.43	1.95	1.59	3.74	3.55	1.39	− 4.078	< 0.001**
22	UB−	2.70	3.30	1.96	3.90	4.75	1.85	− 2.536	0.011**
23	UB+	3.79	4.00	2.64	5.21	5.05	2.69	− 4.079	< 0.001**
24	OL−	8.89	8.10	5.71	10.52	10.15	5.64	− 4.111	< 0.001**
25	OL+	4.16	4.30	2.55	5.61	5.25	2.77	− 2.527	0.012**
26	TT−	5.44	4.80	2.05	7.56	6.85	2.12	− 2.521	0.012**
27	TT+	8.95	8.30	4.38	10.98	10.35	4.31	− 4.110	< 0.001**
28	TS−	5.74	5.10	1.63	8.11	7.70	1.32	− 2.530	0.011**
29	TS+	8.44	8.35	4.99	10.44	10.20	4.83	− 4.113	< 0.001**
30	KNM−	6.29	7.50	3.48	8.38	8.80	3.56	− 4.024	< 0.001**
31	KNM+	3.62	3.40	2.36	5.78	5.60	2.47	− 2.677	0.007****
32	KSM−	3.19	2.45	2.78	4.66	4.20	2.89	− 2.524	0.012**
33	KSM+	5.68	5.50	2.87	7.95	7.75	2.67	− 4.110	< 0.001**
34	UK−	2.69	1.50	2.15	4.79	4.05	2.05	− 2.527	0.012**
35	UK+	8.03	6.95	5.33	9.74	9.20	5.23	− 4.110	< 0.001**

*Source*: Own research

**p < 0.01

**Table 3:** Significance of differences in the value of posture features in the frontal plane between 1st and 2nd measurement with the back loading among girls

No	Variables	Measurement 1			Measurment 2			Wilcoxon’s test	
		M	Me	SD	M	Me	SD	Z	*p*
1	DCK	308.98	314.05	22.87	290.72	292.65	21.15	− 4.782	< 0.001**
2	Alfa	8.28	8.45	1.52	4.82	4.85	2.10	− 4.783	< 0.001**
3	Beta	9.90	9.75	1.13	21.76	21.70	1.17	− 4.785	< 0.001**
4	Gamma	11.10	11.20	1.19	9.10	9.00	1.16	− 4.788	< 0.001**
5	Delta	29.28	29.65	2.45	35.69	35.40	2.70	− 4.785	< 0.001**
6	KPT−	3.74	4.15	1.34	5.72	6.40	1.59	− 3.929	< 0.001**
7	KPT+	4.40	4.75	0.69	15.43	16.50	2.32	− 2.807	0.005**
8	DKP	278.15	279.00	8.96	269.48	273.10	20.01	− 4.785	< 0.001**
9	KKP	159.04	159.00	1.55	149.14	148.90	1.63	− 4.785	< 0.001**
10	RKP	185.63	185.30	13.49	181.28	180.50	13.68	− 4.389	< 0.001**
11	GKP	20.26	19.95	1.40	15.43	15.35	1.69	− 4.786	< 0.001**
12	DLL	246.61	247.00	11.98	242.15	242.90	12.20	− 4.785	< 0.001**
13	KLL	161.82	161.95	2.22	153.42	153.35	2.52	− 4.784	< 0.001**
14	RLL	134.86	135.60	11.07	130.68	131.55	10.78	− 4.785	< 0.001**
15	GLL	23.44	24.45	3.19	32.84	32.65	3.36	− 4.788	< 0.001**
16	KNT−	1.56	1.40	1.04	2.61	2.25	1.03	− 4.116	< 0.001**
17	KNT+	2.04	2.35	1.50	3.83	4.10	1.88	− 2.524	0.012**
18	KLB−	2.60	1.90	1.64	4.31	4.00	1.31	− 2.527	0.012**
19	KLB+	1.60	1.05	1.39	3.02	2.70	1.59	− 4.112	< 0.001**
20	UL−	3.01	4.15	2.30	4.35	5.30	2.29	− 2.527	0.012**
21	UL+	2.43	1.95	1.59	3.74	3.55	1.39	− 4.078	< 0.001**
22	UB−	2.70	3.30	1.96	3.90	4.75	1.85	− 2.536	0.011**
23	UB+	3.79	4.00	2.64	5.21	5.05	2.69	− 4.079	< 0.001**
24	OL−	8.89	8.10	5.71	10.52	10.15	5.64	− 4.111	< 0.001**
25	OL+	4.16	4.30	2.55	5.61	5.25	2.77	− 2.527	0.012**
26	TT−	5.44	4.80	2.05	7.56	6.85	2.12	− 2.521	0.012**
27	TT+	8.95	8.30	4.38	10.98	10.35	4.31	− 4.110	< 0.001**
28	TS−	5.74	5.10	1.63	8.11	7.70	1.32	− 2.530	0.011**
29	TS+	8.44	8.35	4.99	10.44	10.20	4.83	− 4.113	< 0.001**
30	KNM−	6.29	7.50	3.48	8.38	8.80	3.56	− 4.024	< 0.001**
31	KNM+	3.62	3.40	2.36	5.78	5.60	2.47	− 2.677	0.007****
32	KSM−	3.19	2.45	2.78	4.66	4.20	2.89	− 2.524	0.012**
33	KSM+	5.68	5.50	2.87	7.95	7.75	2.67	− 4.110	< 0.001**
34	UK−	2.69	1.50	2.15	4.79	4.05	2.05	− 2.527	0.012**
35	UK+	8.03	6.95	5.33	9.74	9.20	5.23	− 4.110	< 0.001**

*Source*: Own research

**p < 0.01

**Fig. 3:**
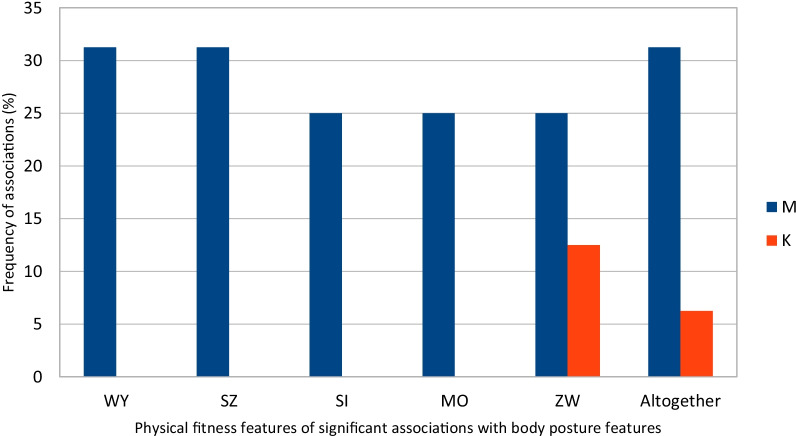
The frequency of significant associations of physical fitness features in the frontal plane with the body posture features among 7-year-old boys and girls n = 65. The legend WY: endurance, SZ: speed, SI: strength, MO: power, ZW: agility. Altogether—the percentage of physical fitness features of significant associations with body posture features. M—boys, K—girls

**Fig. 4:**
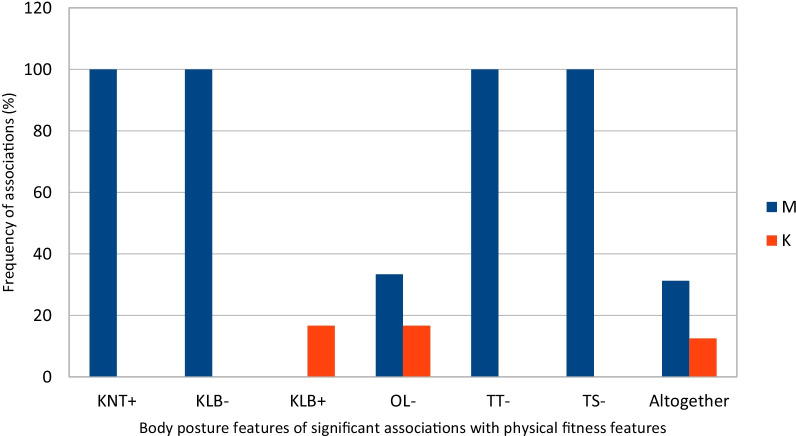
The frequency of significant associations of body posture features in the frontal plane with physical fitness features among 7-year-old boys and girls n = 65. The legend WY: endurance, SZ: speed, SI: strength, MO: power, ZW: agility. Altogether—the percentage of physical fitness features of significant associations with body posture features. M—boys, K—girls

The physical fitness presented by children and the relationship between its individual elements and the differences in the size of the posture features has a different and gender-dependent meaning. Larger among boys, very small among girls. Among boys, significant and the most common occur with endurance, speed and general fitness, smaller with strength, power and agility, among girls with agility and small with total efficiency, Fig. 3. Among boys, the weight of the backpack significantly and most often disturbs the verticality of the torso (KNT+), the symmetry of the height of the shoulders (KLB−), the distance of the lower shoulder blades angles from the line of the spinous processes of the spine (OL−), the height (TT−) and width (TS−) of the waist triangles, and among girls symmetry of the shoulder height (KLB+) and the distance of the lower shoulder blades angles from the line of the spinous processes of the spine (OL−), Fig. 4.

## Discussion

Analyzing the correlation of differences between the 1st and 2nd measurement and the Sekita’s test results among boys, it turned out that the greater the endurance is, the smaller the differences in the torso bend angle to the right are (KNT+) and the shoulders line angle is, where the left one is higher (KLB−) and the smaller the asymmetry of width of the waist triangles is, where the right one is wider (TS−), and the greater one at a distance from the angles of the lower shoulder blades to the line of the spinous processes of the spine, where the angle of the left shoulder blade is more spaced (OL−) and the height of the waist triangles, where the right one is higher (TT−). The higher the speed is, the smaller the height asymmetry of the waist triangles is, where the right one is higher (TT−), and the greater the differences in the torso bend angle to the right are (KNT+), the angle of the shoulders line, where the left one is higher (KLB−) and the greater the asymmetry in the distance of the lower angles of the shoulder blades from the line of the spinous processes of the spine, where the angle of the left shoulder blade is more spaced (OL−) and the width of the waist triangles where the right one is wider (TS−). The greater the strength is, the smaller the differences in the angle of the torso bend to the right are (KNT+), the angle of the shoulders line where the left one is higher (KLB−), and the smaller the asymmetry in the width of the waist triangles is, where the right one is wider (TS−) and the greater the height of waist triangles is where the right one is higher (TT−). The greater the power is, the smaller the differences in the torso bend angle to right are (KNT+), the shoulders line angle, where the left one is higher (KLB−), the smaller the asymmetry in the width of the waist triangles is, where the right one is wider (TS−), and the greater the height of waist triangles is, where the right one is higher (TT−). The greater the agility is, the smaller the differences in the angle of the torso bend to the right are (KNT+), the angle of the shoulders line, where the left one is higher (KLB−), the smaller the asymmetry of the width of the waist triangles is, where the right one is wider (TS−), and the greater the height of the waist triangles is, where the right one is higher (TT−). The greater the general fitness is, the smaller the differences in the torso bend angle to the right are (KNT+), the angle of the shoulders line, where the left one is higher (KLB−) and the smaller the asymmetry in the width of the waist triangles is, where the right one is wider (TS−) and the greater the height of the waist triangles where the right one is higher (TT−), Table 4. The correlation analysis among girls shows that the greater the agility is, the smaller the difference in the angle of the shoulders line is, where the right shoulder blade is higher (KLB+), and the greater the asymmetry of the distance of the lower shoulder blades angles from the line of the spinous processes of the spine is, where the angle of the left shoulder blade is more spaced (OL−), Table 5.

**Table 4:** Correlations between physical fitness and the difference in the value of posture features in the frontal plane between the 1st and 2nd measurement with the back loading among boys n = 30

Variables	The difference between 1st and 2nd measurement					
	WY	SZ	SI	MO	ZW	OG
KNT−	0.25	0.27	0.18	0.30	0.13	0.24
KNT+	− 1.00**	1.00**	− 1.00**	− 1.00**	− 1.00**	− 1.00**
KLB−	− 1.00**	1.00**	− 1.00**	− 1.00**	− 1.00**	− 1.00**
UL−	0.23	0.14	0.51	0.22	− 0.14	0.13
OL−	0.71**	0.58*	− 0.05	− 0.24	− 0.07	0.09
OL+	0.24	0.17	0.10	− 0.50	0.18	0.11
TT−	1.00**	− 1.00**	1.00**	1.00**	1.00**	1.00**
TT+	0.43	0.47	0.13	− 0.46	0.17	0.22
TS−	− 1.00**	1.00**	− 1.00**	− 1.00**	− 1.00**	− 1.00**
TS+	− 0.26	− 0.01	0.44	0.50	− 0.02	0.31
KNM−	− 0.04	− 0.15	− 0.04	0.36	− 0.15	− 0.04
UK−	0.10	− 0.18	0.21	0.22	0.17	− 0.20

*Source*: Own research

**p < 0.01

**Table 5:** Correlations between physical fitness and the difference in the value of posture features in frontal plane between the 1st and 2nd measurement with the back loading among girls n = 35

Variables	The difference between 1st and 2nd measurement					
	WY	SZ	SI	MO	ZW	OG
KLB+	− 0.15	− 0.08	− 0.70	0.56	− 0.79*	− 0.53
UL+	− 0.21	− 0.45	− 0.14	− 0.46	− 0.08	− 0.33
OL−	0.16	0.00	0.67	− 0.42	0.76*	0.54
TT+	0.47	0.61	0.22	0.00	0.31	0.29
TS−	− 0.50	0.20	− 0.16	− 0.22	− 0.30	− 0.30
TS+	0.22	0.30	− 0.26	0.18	− 0.20	− 0.11
KNM+	0.74	0.05	0.36	0.35	0.53	0.53

*Source*: Own research

*p < 0.05

Romanowska [24] and Mrozkowiak [12], based on the results of studies in a smaller group of adolescents, attempted to describe changes influenced by the student's posture loaded with an external load. The authors in their investigations came to very similar conclusions. The six-kilogram symmetrical load of the upper limb girdle in 12-year-old girls caused no significant changes in the value of selected posture features. Mrozkowiak [12] showed a complete restitution of the value of the diagnosed features two minutes after the load removal. The return to the initial value after the first minute was more intense. The author also concluded that symmetrically distributed load has little effect on the spine-pelvic syndrome in the frontal plane, including right-hand scoliosis at the Th3 level. Mrozkowiak [25] in his research on the effects of loading with school supplies in the left or right hand drag mode of the body posture in the frontal plane of a 7-year-old student showed that the increased load causes significant changes in the value of selected body posture features among girls and boys. He believes that the greater the weight of the container, the carrying time and the intensity of manual effort is the grater the changes will be. Therefore, this way of carrying school supplies by first-grade students should not be recommended. He also proved that there is a various relationship with the values of changes in body posture with the level of general physical fitness. This relationship is more common among boys than among girls and only with the right hand drag mode. Considering individual abilities, among boys there are relationships with significant differences in the value of posture features like speed, power, endurance and agility, and among girls there is strength additionally. Mrozkowiak [26] made a different analysis of the same results to answer the question, which of the ways of carrying disturbs the child's habitual posture less? It turned out that there was simultaneously significant and negative disturbance in the posture stability caused by the left and right hand drag mode. The author believes that it may cause disorders and, consequently, defects in body posture in the long term. Therefore, neither of them should be recommended. The author also claims that general physical fitness has a greater positive significance in disorders of biomechanical body posture statics among boys than girls. Its relationships with particular features are similar in both modes of carrying among boys, whereas among girls greater relationships occur in the case of right-hand drag mode. The most significant motor skills among boys are endurance and strength, and among girls, speed and power. The restitution value of any of the analyzed features of body posture was not complete after 1 and 2 min when right or left hand drag mode stopped. This proves insufficient physical fitness, its laterality and slower restitution. The author's survey among parents of 7-year-old preschoolers shows there is the guardians’ awareness about their children's health. They believe that a first grader will carry a four-kilogram schoolbag on their back, learn traditionally (not via online learning) and spend about 2 h improving their physical fitness. According to the author, the accepted lifestyle will not improve physical fitness and prevent statics posture disorders [23].

According to Grimmer et al. [27] carrying various loads by school children and adolescents can result in fatigue, muscle pain, back and shoulder pain, hand numbness, and in extreme cases, spine injury [25]. Research by Skoffer [28] and Grimmer et al. [29] showed that 50% of teenagers feel pain in the spine, which is caused by carrying various containers with school supplies. Studies by Negrini and Negrini [30], Pau and Pau [31] and Heler et al. [32] have shown that children’s postural stability disrupted with an additional load in the form of a backpack may lead to impaired postural control, thus increase the risk of falls and an advance occurrence of back pain. The results of Pau's work indicate significant changes on postural features influenced by additional load in terms of swaying, the maximum range of deflection, the length of the COP path (center of foot pressure) and the posturogram envelope [31].

Adams' research indicates a significant influence of the shifted tightening loads on changes occurring in a single kinesthetic segment of the spine [33]. Pain associated with carrying a backpack are known as the so-called backpack syndrome. The syndrome includes the following factors: abnormal body posture causing headaches, fatigue, pain in the cervical and lumbar spine, and increased muscle tension in the neck, shoulders and back [34, 35].

The statistical analysis of the obtained measurements of selected posture features clearly shows that the method of transporting a backpack with school accessories weighing more than 4 kg should not be practiced by 7-year-old children because it significantly disturbs its habitual stability in the frontal plane. It should be assumed that the longer and more intensive the analyzed mode of transport, and the greater the mass of utensils, the more significant the adaptive changes will be. The age of the surveyed students is also important.

Przeprowadzone badania pozwoliły określić wpływ obciążenia przenoszonego na plecach, na postawę ciała badanych dzieci. Wartością badania było wykorzystanie metody fotogrametrycznej jako jednej z bardziej obiektywnych i nieinwazyjnych metod diagnozowania postawy ciała. Na potrzeby badania wykorzystano autorskie narzędzie diagnostyczne do oceny postawy ciała—opatentowaną ramę. Wyjątkowość badania dotyczyła również pomiarów wielkości cech opisujących postawę ciała po usunięciu obciążenia zewnętrznego. Ograniczeniem badań były zbyt krótki okres badawczy oraz niewielka ilość badanych dzieci. Korzystne byłyby dalsze badania przed początkiem roku i po zakończeniu rocznego cyklu szkoleniowego.

## Conclusions

1. Carrying school supplies on the back causes significant changes in the value of the features describing the body posture in the frontal plane. The differences were greater among girls than among boys.
2. Physical fitness has a diversified and sex-dependent influence on the value of the changes in body posture features under the influence of the adopted carrying of school supplies. Among boys it significantly affects the asymmetry of the torso bend, shoulder height, height and width of waist triangles, and among girls the asymmetry of the shoulders and the distance of the lower angles of the shoulder blades from the line of the spinous processes of the spine. Among boys, endurance and speed influence the changes in the value of the body posture features the most, but strength, power and agility less, whereas, among girls only agility matters.
3. It should assumed that the greater the weight of the container, the transport time and the intensity of the effort physical is, the greater the changes will be. Therefore, carrying school supplies should not weigh more than 4 kg by first-graders.

## Data Availability

The datasets used and/or analyzed during the current study are available from the corresponding author on reasonable request.
